# Androgen Receptor mRNA levels determine the prognosis in triple-negative breast cancer patients

**DOI:** 10.1186/s12885-020-07218-0

**Published:** 2020-08-10

**Authors:** Sindhu Govindan, Mallikarjuna Siraganahalli Eswaraiah, Chetana Basavaraj, Manjula Adinarayan, Satish Sankaran, Manjiri Bakre

**Affiliations:** OncoStem Diagnostics Private Limited, # 4, Raja Ram Mohan Roy Road, Aanand Tower, 2nd Floor, Bangalore, Karnataka 560027 India

**Keywords:** TNBC, Androgen Receptor, Prognosis, FOXA1, GATA3, qRT-PCR

## Abstract

**Background:**

Anti-Androgen Receptor (AR) therapy holds promise for a subset of AR expressing triple-negative breast cancer (TNBC) patients. However, current AR assays are suboptimal in detecting the dynamic range of AR expression, contributing to its controversial role in TNBC disease prognosis. This study is aimed at evaluating the feasibility of qRT-PCR to sensitively and robustly detect AR mRNA levels for prognostication.

**Methods:**

mRNA expression profiling was performed on FFPE blocks from a retrospective cohort of 101 TNBC patients using qRT-PCR and compared with AR protein expression by immunohistochemistry . Statistical analyses included Spearman’s rank correlation, Chi-square and Kaplan-Meier analyses. Distant Metastasis Free Survival was used as the end point in survival analysis.

**Results:**

AR mRNA expression was observed in 34/101 patients (34%) whereas 12/80 cases (15%) were positive by IHC. qRT-PCR could thus detect more AR positive patients as compared to IHC, with 75% (9/12) concordance between the two methods. Co-expression of GATA3 and FOXA1 mRNA was observed in 85 and 88% of AR mRNA positive tumors, respectively. AR mRNA positivity was significantly correlated with age at disease onset (*p* = 0.02), high FOXA1/GATA3 (*p* < 0.05) and distant recurrence. AR mRNA positive patients had poorer DMFS (43%; *p* = 0.002). DMFS dropped further to 26% (*p* = 0.006) in AR (+)/high FOXA1/GATA3 patients. AR mRNA expression together with node positivity had the worst DMFS (23%; *p* < 0.0001) compared to patients who were either positive for any one of these, or negative for both AR and node status. Low Ki67 mRNA with AR mRNA positivity also had poorer DMFS (39%; *p* = 0.001) compared to patients expressing low Ki67 with no AR mRNA expression.

**Conclusion:**

qRT-PCR was more sensitive and reliable in detecting the dynamic expression levels of AR compared to IHC and this variation could be explained by the higher sensitivity of the former method. High AR mRNA expression was strongly associated with expression of AR protein, high FOXA1/GATA3 mRNA, and with poor prognosis. qRT-PCR was more efficient in detecting the AR positive cases compared to IHC. A distinct signature involving high GATA3/FOXA1, low Ki67, and node positivity in AR mRNA positive tumors correlated with poor prognosis. Thus, AR mRNA screening can serve as an effective prognostic marker along with offering potential targeted therapy options for TNBC.

## Background

The incidence of triple-negative breast cancer (TNBC) varies from 6.7 to 27.9% in different countries, with the highest percentage reported in India [1]. TNBC exhibits high intra-tumoral heterogeneity and its distinct molecular features contribute to a varied treatment response. The molecular type is thus important for guiding clinical treatment and evaluating prognosis. Molecular classification has identified seven TNBC subtypes, including the luminal AR (LAR) subtype characterized by androgen receptor (AR) expression [2]. However, none of the molecular subtyping signatures are translated into commercial prognostic assays in TNBC due to lack of strong validation in independent data sets. Though multi-gene signatures have been increasingly developed and used for breast cancer prognosis, studies show that expression of a single critical gene can also serve as prognostic indicator for specific tumor subtypes [3]. AR expression has been extensively studied but has been a controversial biomarker to predict TNBC prognosis [4–6]. While many immunohistochemistry (IHC) based studies show AR expression in TNBC as an indicator of better prognosis [7, 8], a few recent reports (also IHC based) also show an inverse correlation with prognosis [4, 9, 10]. This inconsistency impedes the clinical utility of this marker for prognosis in TNBC. If the prognostic role of AR could be successfully validated, it can be used as potential therapeutic target in TNBC as it is a subtype without suitable targets. Since AR expressing tumors are less chemo-responsive in nature, it can also be used to choose patients for neoadjuvant therapy.

A phase II trial (MDV3100–11) has demonstrated the clinical utility of the AR antagonist enzaliutamide in a large cohort of locally advanced AR-positive TNBC [11]. Thus, in addition to prognosis, AR expression provides alternative therapy options for TNBC patients for whom chemotherapy/radiotherapy is the only default option. Most published studies have used) IHC as the detection method for AR expression. However, factors like antibody clone, IHC protocol, and detection cut-off might lead to differences in outcome correlation across multiple studies. Indeed, these differences could have led to the variability in the results of previous IHC based studies using AR as a prognostic biomarker. In this study we investigated whether a non-subjective, sensitive, and reproducible technique like qRT-PCR can provide a better alternative screening method for accurate testing of AR.

AR is a ligand-binding transcription factor and mediates its effect on cell proliferation and survival through its interaction with FOXA1/GATA3 [2, 12]. Though studies have investigated the prognostic role of all these markers individually [13–16], their combined role in disease prognostication is not well established in TNBC. Current cancer biomarker research has been primarily focusing on transcriptional information and gene regulatory networks to identify more prognostic gene signatures.

We thus explored gene expression profiling of AR and its co-regulatory molecules, such as FOXA1 and GATA3, using real-time qPCR in FFPE specimens of TNBC patients. The prognostic value of these biomarkers was assessed by comparing expression levels with distant metastasis. A concordance analysis between protein and mRNA detection of AR and Ki67 was also carried out to determine method sensitivity.

## Methods

### Patient samples

The study included Formalin-Fixed Paraffin-Embedded (FFPE) tumor specimens from 111 TNBC patients diagnosed with early Stage (I-III) Invasive Ductal Carcinoma (IDC) who underwent surgery followed by adjuvant chemo or radiotherapy. All patients had clinical follow up for a minimum of 4 years. Ethics Committee (EC) approval for the study was obtained from participating hospitals. Informed patient consent was waived as per local guidelines (section 5.7, ICMR Ethical Guidelines, 2017) as the patient cohort in this study were retrospectively selected and the study was non-interventional and anonymized. All the patients were aged between 29 and 75 years, and their ER/PR and HER2 status was e confirmed in our laboratory by IHC. The 4-year complete follow up, and treatment history was collected from hospital records where patients underwent treatment. Exclusion criteria included invasive papillary/adenocarcinoma, patients treated with Neo-Adjuvant Chemotherapy (NACT), patients with incomplete follow up, poor RNA quality and yield. For survival analysis, Distant Metastasis Free Survival (DMFS) was computed based on recurrence at distant sites within 4 years of diagnosis.

### RNA extraction from FFPE tumor blocks

FFPE blocks from surgical specimens that were less than 15 years old, with < 10% necrosis were selected for RNA extraction. Due to the possibility of contamination of tumor RNA with normal tissue RNA, we kept a cut-off of 50% tumor content as a pre-requisite for all samples used in this study. Total RNA was extracted from three curls of 8 μm thick tumor sections using AllPrep DNA/RNA FFPE kit (Qiagen, Germany) as per the manufacture’s protocol. Briefly, the sections were deparaffinized in xylene followed by Proteinase K digestion and in-column DNase treatment. RNA eluted in 30 μl of nuclease-free water was quantified using the RiboGreen fluorescence method on Qubit (Thermo Fisher Scientific, USA). A few samples were checked by Agilent Bioanalyzer (Agilent 2100 Bioanalyzer System, ABI) for RNA integrity number (RIN) check. We found this to be consistently below the normal threshold of RIN 6 or above (1.9–2.6 in our samples). Given that FFPE RNA is often degraded, we preferred to go with the amplification of β-Actin gene as a quality control check for the RNA samples. Residual genomic DNA in the purified RNA samples was assessed by TaqMan quantitative PCR for β-Actin assay (Applied Biosystems, Thermo Fisher Scientific USA). Samples with β-Actin Cq (Cycle quantification threshold) value ≤35 were considered free from DNA contamination and profiled for gene expression.

### Quantitative PCR and analysis of differential gene expression

Total RNA (250 ng–500 ng) was reverse transcribed with High capacity cDNA conversion kit (ABI, Thermo Fisher Scientific, USA) using pooled gene-specific reverse primers (100 nmol/L each, IDT, USA) and random hexamers. Expression of all the reference and target genes were measured in duplicate reactions using cDNA equivalent of 2 ng–8 ng RNA per reaction in Roche light Cycler 480 machine (Roche Diagnostics, Switzerland) using either TaqMan probes (125-250 nM) and primers (200-900 nM) (IDT, USA) or pre-designed assays (Table S1). Amplification efficiencies of the assays (TaqMan assays/probes) used for profiling all the genes were calculated from RNA serial dilution experiments (Supplementary methods) before testing in actual samples [17]. All PCR reactions were subjected to 45 cycles of amplification, and an average Cq of duplicate measurements for each target gene along with the two reference genes (GAPDH and RPLPO) was calculated. Samples that deviated from the group mean Cq (30.3 ± 2.0) of the two reference genes were excluded to compensate for pre-analytical issues. After establishing a comparable amplification efficiency (Fig. S1) for both target and reference genes, reference normalized expression measurements were calculated as the mean Cq of the two reference genes minus the mean Cq of each target gene. Final fold change values were then obtained using the formula, 2^ΔCq*100 and the relative mRNA levels greater than 2.0 were considered as high expression for GATA3, FOXA1, and Ki67. AR expression was categorized as positive or negative based on the presence or absence of amplification signal in PCR reaction.

### Immunohistochemistry of AR and Ki67

AR protein expression was evaluated in 80 of the 101 cases of TNBC using anti-AR monoclonal antibody, (clone AR 441, Thermo Fisher Scientific, USA) on Ventana BenchMark auto stainer system XT (Ventana Medical Systems, USA) as described elsewhere [18]. Briefly, 3 μm thick sections were fixed in a hot air oven at 60 °C for 60 mins and loaded onto the machine for IHC staining of AR. De-paraffinization was done with the EZ Prep solution (Proprietary Ventana reagent), and antigen retrieval was performed using Cell Conditioning solution 1 (CC1) for 64 min. Primary antibody (1:100 dilution in 3% BSA) was added manually and incubated for 1 h at 37 °C. HRP Multimer based OptiView DAB detection kit was used to visualize the signal, utilizing DAB (3–3’diaminobenzidine) as the chromogen. Finally, tissue sections were counterstained with hematoxylin and bluing reagent for 12 min each before removing slides from the autostainer. The stained slides were washed in de-ionized water, dehydrated in graded ethanol, cleared in xylene and evaluated for nuclear staining. Ki67 IHC was done using the pre-diluted Ki67 antibody (Biogenix, Clone Mib1, # AM297-5 M) by manual method as detailed in our earlier publication [19]. Two pathologists graded the percentage of AR and Ki67 positive cells independently. AR positivity was defined by nuclear localization in > 1% of tumor cells [11]. Nuclear Ki67 staining of < 20% was considered low risk, and > 20% was considered high risk [20].

### Statistical analysis

Correlation among gene markers was analyzed by Spearman’s rank correlation coefficient, ‘r’ as a measure of the strength and direction of the linear relationship. The association of AR expression with tumor characteristics was assessed by 2 × 2 contingency table. Median follow up period was calculated for those who were free from events of distant metastases. Kaplan Meier’s survival analysis, Cox proportional hazard ratio (HR) and log-rank test were used to find the prognostic value of all variables. Regression analysis was performed to evaluate co-expression of markers and r values > 0.6 were considered as good correlation. All statistical analyses were performed using MedCalc, and the survival curves were generated using Graph Pad Prism V.3.

## Results

### Study population demography

**Fig. 1:**
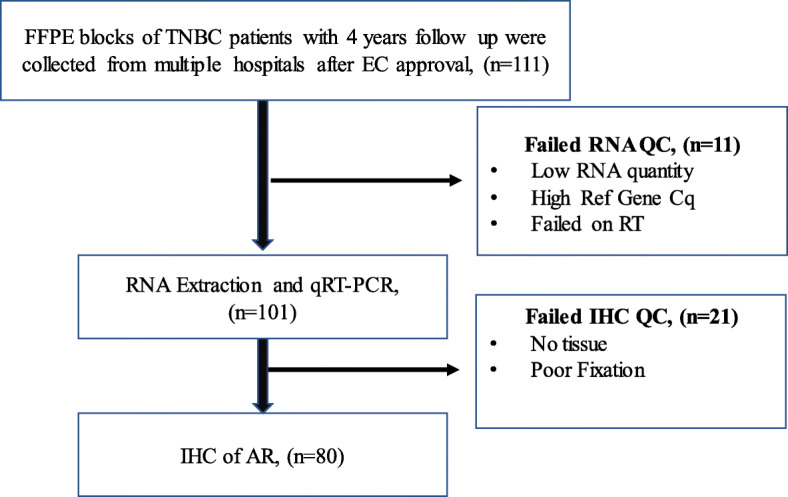
Flow chart of selection of study population. FFPE, Formalin Fixed Paraffin Embedded; EC, Ethical Committee; QC, Quality Check; TNBC, Triple- negative breast cancer; IHC, Immunohistochemistry; qRT-PCR, quantitative RealTime PCR; RT, Reverse Transcription

The study cohort included 111 TNBC patients who were negative for ER/PR and HER2, as confirmed by IHC. Of these 111 samples, 101 FFPE samples qualified for qRT-PCR as shown in the study flow chart (Fig. 1), 10 samples with poor RNA quality and quantity (insufficient RNA quantity, genomic DNA contamination, failed in reverse transcription) were excluded from qRT-PCR analysis. Eighty of these 101 patients qualified for AR IHC, twenty-one samples were excluded from IHC analysis due to lack of tissue and/or fixation issues. The clinicopathological characteristics of the patients are summarized in Table 1. Median age at onset of patients was 51.0 years (range 29–75) with majority of them (72%) in the 40–60 years range. In this cohort, there was equal distribution (50%) of node-negative and positive patients. A majority of these tumors were of stage II (75%) with histological grade 2 (40%) and 3 (53%). The median follow-up period for those who were event-free was 59 months. The median time to the first recurrence, which was considered as time to progression (TTP), was 24 months with a range of 4–48 months.

**Table 1:** Clinical and tumor characteristics of the study cohort

Parameter	Number (***n*** = 101)	Frequency (%)
**Age**		
< 40	13	13.0
40–60	72	71.0
> 60	16	16.0
**Tumor Grade**		
1	7	7.0
2	41	41.0
3	53	53.0
**Node status**		
Negative	50	50.0
Positive	51	50.0
**Tumor Stage**		
I	3	3.0
II	76	75.0
III	22	22.0
**Recurrence**		
No recurrence	64	63.0
Recurrence	37	37.0

### Relative mRNA levels of markers and their co-expression

**Fig. 2:**
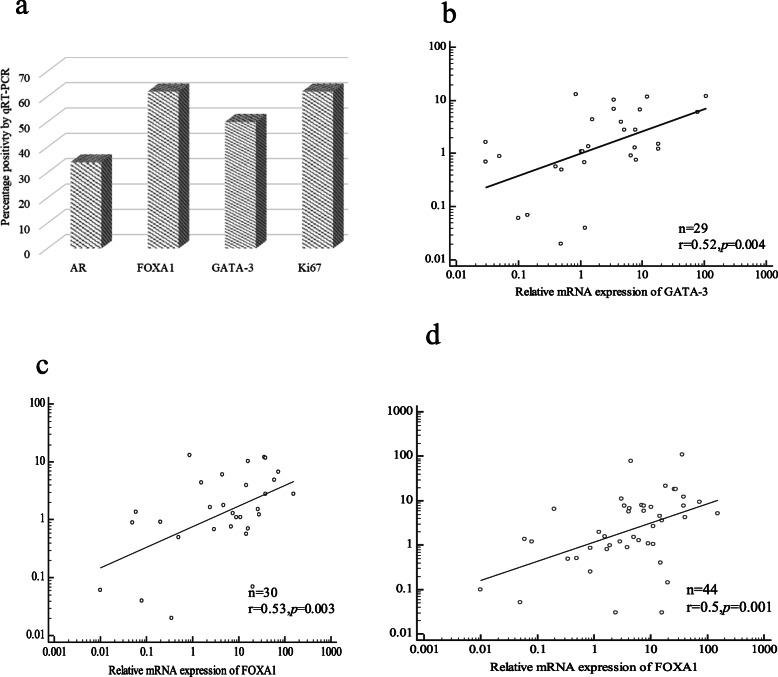
Expression AR, GATA3, FOXA1 and Ki67 mRNA levels in total cohort. The frequency distribution of each gene in the study cohort is shown in Bar graph (**a**). Linear regression analysis was done to find the co-expression of AR, GATA3 and FOXA1 using the direct fold expression values. The scatter plot shows a significant co-expression of AR with GATA3 (**b**), FOXA1 (**c**) and between FOXA1 and GATA3 (**d**). The correlation co-efficient, r was ≥0.5 and significant (*p* < 0.05) for all combinations

 AR expression by qRT-PCR was observed in 34 of 101 (34%) TNBC tumors profiled. Expression of GATA3, FOXA1, and Ki67 mRNA were detected respectively in 50, 62, and 61% of the patients in the cohort (Fig. 2a). AR was found to be co-expressed with its downstream regulators, GATA3 and FOXA1 by regression analysis (Spearman’s rank correlation) using the continuous expression values. There was a moderate linear correlation of AR mRNA level with GATA3 (r = 0.52; *p* = 0.004, *n* = 29) and FOXA1 (r = 0.53; *p* = 0.003, *n* = 30) mRNA expression (Fig. 2b, c). Likewise, mRNA levels of GATA3 and FOXA1 also showed a significant linear correlation (r = 0.5, *p* = 0.001, *n* = 44) (Fig. 2d). However, analysis of different expression thresholds of AR mRNA showed that higher expression (> 1 fold cut off) was associated with high FOXA1 and GATA3 mRNA levels. Conversely, FOXA1 and GATA3 mRNA expressions were low when AR mRNA was low (≤1 fold cut-off) (Table S2). In the AR (+) subgroup, 63 and 44% of cases were double-positive for AR (+)/FOXA1 (+) and AR (+)/GATA3 (+) phenotypes respectively. In the AR (+) cohort, 35% patients were triple-positive with an AR (+)/GATA3 (+)/FOXA1(+) signature.

Furthermore, a majority (71%, 24/34) of AR (+) tumors had low or no Ki67 mRNA expression. IHC analysis for Ki67 could only be performed in 21 of these. Seven had low Ki67 using a 20% cut off, and in all, 62% (13/21) concordance was observed between Ki67 IHC results and Ki67 mRNA expression (Fig. S2).

### Correlation between AR protein and mRNA expression

**Fig. 3:**
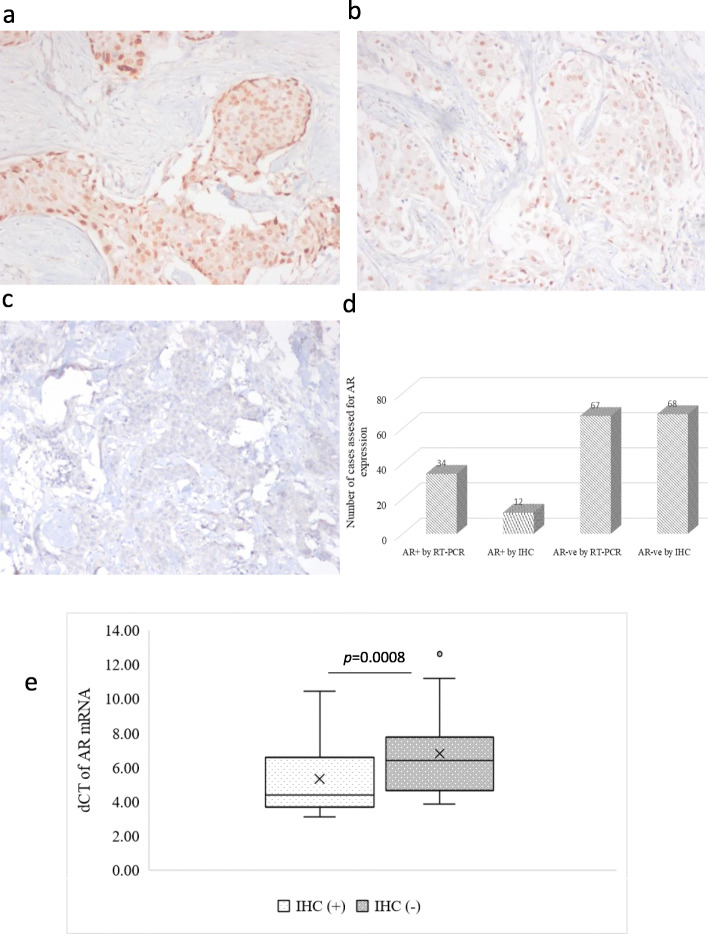
Immunohistochemistry analysis of AR and comparison with qRT-PCR positivity. IHC was carried out in the tumor specimens (*n* = 80) using anti human mouse monoclonal AR antibody and the percentage of nuclear stained cells were graded for AR positivity. Representative images were shown for strong AR expression (**a**), weak expression (**b**) and negative staining (**c**). Bar graph showing the number of cases assessed for AR expression by two methods (IHC and qRT-PCR) of detection (**d**). The whisker plot showing a comparison of the delta Cq values of AR IHC (+) tumors and AR IHC (−) tumors. AR IHC (+) cases had low delta Cq values indicating high AR mRNA expression and AR IHC (−) cases had high delta Cq values indicating a low expression of AR (**e**). All the images were captured with a magnification of 100x

As mentioned above, AR protein expression could be evaluated by IHC in 80 out of 101 samples, and 15% (12/80) were found to be AR positive. The percentage of cells with nuclear staining in these AR positive samples varied between 10 to 95% (Fig. 3a-c). We observed concordance between AR immune-positivity and AR mRNA expression in 75% (9/12) of cases (Table S3) and 3 cases were negative by qRT-PCR. The results remained consistently negative for the PCR negative but IHC positive samples, even after multiple repeats. Nonetheless, the concordance measured between AR mRNA and protein expression at different thresholds of AR mRNA cut off (< 1, 1–10 and > 10 fold), revealed that the correlation was more significant at > 10 fold expression with 100% concordance and less significant at < 1 fold, with only 30% concordance (Table S4). However, by qRT-PCR, 18 additional samples were positive for AR mRNA expression (Fig. 3d), of which 44% (8/18) had AR mRNA lower than 1 fold. We found a difference in the transcript levels of AR mRNA between IHC positive and IHC negative cases (Fig. 3e). The IHC (−)/qRT-PCR (+) samples (*n* = 18) had significantly lower levels of AR mRNA as observed from their higher delta Cq values as compared to IHC (+)/qRT-PCR(+) samples with lower delta Cq values and higher expression (Fig. 3e). Furthermore, we investigated the correlation between AR protein expression and prognosis in AR (+) subgroup by IHC. We found that patients with AR protein expression had a better DMFS (84%) compared to those with no AR expression (65%), though it was statistically not significant (Fig. S3).

### Association between AR mRNA levels and clinicopathological features

To understand the clinical application of AR expression, patients were stratified based on AR detectionby qRT-PCR, as positive and negative. AR mRNA positivity by qRT-PCR was further correlated with clinicopathological features and other tumor biomarkers as shown in Table 2. The analysis showed an association between AR positivity and patient age. We found a significant positive correlation between age at disease diagnosis and AR expression (*p* = 0.02). The percentage of AR (+) patients also increased with increase in the age of patients. No significant association was observed between AR mRNA expression with tumor size, nodal status, tumor stage, and Ki67 status. There was a strong positive correlation between AR positivity and FOXA1 (*p* = 0.03) and GATA3 (*p* = 0.005) mRNA expression levels. AR (+) mRNA expression significantly increased the likelihood of disease recurrence in this cohort as observed from their correlation with distant metastasis. AR (−) patients had less likelihood of distant metastasis over four years of follow up period (Table 2).

**Table 2:** Association of clinicopathological features with AR by χ2 test

Variables	All	% of AR (−)	% of AR (+)	***p***-value
**Age**				**0.02**
< 40	13	92	08	
40–60	72	67	33	
> 60	16	44	56	
**Tumor Grade**				0.08
1	7	29	71	
2	41	66	34	
3	53	72	28	
**T- size**				0.57
1–2	86	67	33	
> 2	15	60	40	
**Node status**				0.72
N0	50	68	32	
N+	51	65	35	
**Tumor Stage**				0.41
I	3	67	33	
II	76	70	30	
III	22	55	45	
**Ki67**				0.55
Low/No	75	68	32	
High	26	62	38	
**FOXA1**				**0.03**
Low	54	76	24	
High	47	55	45	
**GATA3**				**0.005**
Low	74	74	26	
High	27	44	56	
**Distant metastasis**				**0.004**
No	64	77	23	
Yes	37	49	51	

### Prognostic importance of AR expression in TNBC patients

**Fig. 4:**
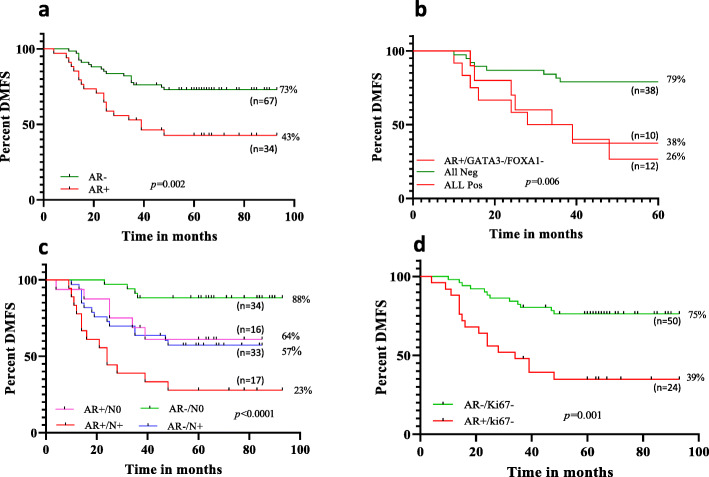
Kaplan Meier survival curves of TNBC patients. **a** Survival analysis in total of 101 patients based on AR expression. AR (+) patients had shorter DMFS than AR negative patients. **b** Distant metastasis-free analysis of patients according to AR/FOXA1/GATA3 status. **c** Distant metastasis-free analysis of patients according to AR and Node stratification, **d** Distant metastasis-free analysis of patients according to AR and Ki67 status

The prognostic analysis in TNBC patients was carried out by both survival analysis and Cox proportional hazard ratio (HR) with distant metastasis as an event. Within the follow-up period of 4 years, 37% (37/101) distant metastasis events were observed (Table 2). Kaplan-Meier survival analysis showed that AR positivity was significantly associated with poor prognosis (DMFS of 43%, *p* = 0.002) (Fig. 4a). In AR (+) TNBC patients, the DMFS was 30% lesser compared to the AR (−) patients (Fig. 4a). Since AR expression was found to be strongly associated with FOXA1 and GATA3 beyond a cut off level ≥ 1 fold, we also analyzed the clinical outcome of TNBC patients using different expression levels of AR mRNA. DMFS for AR no/low (< 1 fold; DMFS of 71%) patients was high compared to that of AR high (> 1 fold; DMFS of 35%; *p* = 0.002) patients (Fig. S3b). We thus observed a further drop in the percentage DMFS by 8% in AR high patients compared to AR (+) tumors (DMFS of 43%) (Fig. S3b and Fig. 4a). We did not observe independent correlations of GATA3 and FOXA1 with prognosis (data not shown). We further analyzed the prognosis in patients who are triple positive (AR+ and high FOXA1/ GATA3). In TNBC patients, expression of all the three markers was significantly related to poor DMFS (26% DMFS, *p* = 0.006) compared to the patients who were negative for all these markers (79% DMFS), (Fig. 4b). Interestingly, the DMFS of patients positive for all three markers (AR+, GATA3 high, and FOXA1 high) was lower than when AR alone [AR (+)/FOXA1(−)/GATA3(−)] was positive (26% vs. 38%) making the combination a more significant predictor of poor prognosis than AR positivity alone. By univariate analysis, age (HR 1.99, 95% CI 1.09–3.61*, p* = 0.02), tumor stage (HR 5.3, 95% CI 2.7–9.9*, p* < 0.001), and levels of (high vs. low) AR expression (HR 6.6, 95% CI 1.4–31.17*, p* = 0.016) were significant predictors for distant metastasis (Table S5). However, with multivariate analysis, only tumor stage (HR 7.3, 95% CI 1.1–48.35*, p* = 0.038) and AR expression levels (HR 8.47, 95% CI 1.5–45.5*, p* = 0.012) were significant (Table S5) predictors of DMFS.

### AR mRNA expression increases the risk of recurrence in patients with node positivity and indolent tumors

To understand the significance of AR mRNA expression in lymph node positive patients, DMFS analysis of AR and node status was carried out. When AR status [AR (+) or (−)] was combined with node status (N+ or N0), DMFS of AR(+)/N(+) patients were significantly lower (23%; *p* < 0.0001, (Fig. 4c)) as compared to all other patients. Patients who were node negative (N0) with no AR expression had a higher survival rate (DMFS of 88%). However, patients with either AR (+) or node positivity had a moderate risk of distant metastasis (DMFS of 64–57%).

Similarly, the association between AR expression and Ki67 with clinical outcome was analyzed. It was found that AR expression in low proliferative (low/no Ki67 mRNA expression) tumors was associated with poorer prognosis (DMFS of 39%, *p* = 0.001) as compared to low proliferative tumors with no AR expression (DMFS of 75%) (Fig. 4d).

## Discussion

We have previously used a combination of biomarkers, clinical parameters and a machine learning algorithm to develop CanAssist Breast, a cost-effective prognostic test for hormone positive breast cancer [21–24]. The current study is an exploratory step to develop a similar prognostic test for TNBC. Although AR has been explored as a prognostic marker in TNBC [4–8, 10], these IHC based studies have shown inconsistent conclusions. Some of these studies have also found no association of AR with DMFS or overall survival (OS) [5, 25, 26]. These inconclusive findings could be explained by the potential variations due to differences in detection methods and/or the difference in the cut-offs selected for defining AR positivity (1 to 10%). Our results indicate that qRT-PCR based detection of AR mRNA would be a better prognostic approach. Such an improved quantification method could help in better stratification of TNBC patients for AR directed therapies [27–29].

There are very few studies that have investigated AR mRNA expression in BC patients and none of these studies probed into correlation of mRNA expression with disease prognosis [30, 31]. In most published studies, AR positivity has been defined as ≥10% tumor nuclei staining [28, 32] by IHC. Attempts have been made to optimize the AR IHC assay using different antibody clones and varying cut-off values to increase the sensitivity and specificity [11, 18]. Subsequently, a Phase II clinical trial (MDV3100–11) for advanced TNBC patients, evaluating the response to enzalutamide treatment, used 0% cut-off for treating patients who were AR positive. This low threshold cut-off was selected to increase accrual rates and maximize the number of patients who could benefit from anti-AR therapy [11]. Based on this study, we used a cut-off of 1% nuclear staining for IHC and positive amplification signal by qRT-PCR for defining AR positivity. Our results showed 15% positivity by IHC and 34% positivity by qRT-PCR, indicating the latter method is more sensitive than IHC. We have also correlated AR protein expression to mRNA levels to evaluate concordance between the two methods. We found 75% concordance between AR protein and mRNA levels by IHC and qRT-PCR respectively. The concordance was more prominent beyond the > 10 fold cut off for AR mRNA expression level indicating the lack of detection sensitivity below this threshold by IHC. This also suggests a mediocre sensitivity of IHC in detecting lower levels and has resulted in non-concordance between two techniques in 25% cases. Protein detection in about 30–33% cases with < 10 fold AR mRNA rules out the possibility of artifacts/false positivity by qRT-PCR. Moreover, the proportion of AR (+) patients by IHC was also lower (15%) compared to other studies that used IHC as a method of detection [33, 34] despite using the most common diagnostic clone (AR441) for AR IHC in our study. We propose that, the discordance in three cases wherein qRT-PCR failed to amplify the AR mRNA but positive by IHC might be either due to rapid degradation of transcribed mRNA or other technical reasons such as differences in the AR mRNA sequences recognized by probe and antibody. The TaqMan assay probe binding region was towards the 3′ region (Exon 4–5) of AR mRNA whereas the epitope of the commercially available clone of AR antibody was designed in the 5′ region (Exon 1). Despite the difference in the binding site, the sequence for both primer and antibody binding region are conserved across all the variant isoforms of AR and hence might rule out the possibility of not detecting any truncated/mutated isoform. Such kind of discordance between detectable levels of mRNA and its corresponding protein has been reported for ER in other studies [35].

Our study also found correlation between AR expression and prognosis to be dependent on the method of detection used. AR positivity by qRT-PCR correlated with poor prognosis while positivity by IHC correlated with a better prognosis, as reported in previous studies [36, 37]. This discordance was also evident in comparison between Ki67 protein and mRNA expression as there was only 62% concordance between the two methods. The dependence on the method of detection could be explained by a higher sensitivity of qRT-PCR as it minimizes false negative rates. These false negatives resulting from IHC seem justified as tumors with positive protein expression were associated with higher mRNA transcript levels (> 10 fold), and was evidenced from the concordance analysis using various threshold levels of AR mRNA. Similar to our findings, Rangel et al. demonstrated that cases with no AR protein expression also had a lower AR mRNA transcript levels [31].

Results of the correlation analysis of AR mRNA with clinicopathological features revealed that only age and grade of the tumors correlated with AR positivity. Correlation of age with AR positivity has been reported in previous studies by IHC methods [38]. The correlation of tumor grade was not very significant, which could be due to the low number of grade I tumors in our study cohort. The correlation of AR mRNA positive tumors by qRT-PCR tumors with lower-grade tumors has been reported in other studies wherein AR was a favorable outcome predictor [39–42]. We did not observe any significant association between AR mRNA expression with Ki67 mRNA levels or node positivity. However, AR positive but low Ki67 patients had high rate of distant metastases compared to AR (−)/low Ki67 patients. Similarly, AR expression in node (+) was associated with lower metastasis-free survival compared to AR (−)/node (+) patients. Though node positivity itself was an independent predictor of poor prognosis (DMFS of 57%), AR positivity in these tumors further decreased the DMFS (23%). This observation indicated a more aggressive phenotype of node-positive/ AR (+) tumors.

Evidences show that AR expression in TNBC is associated with enrichment of hormone-regulated pathways, including steroid synthesis and androgen/estrogen metabolism [43] similar to estrogen receptor positive breast cancer [44]. Preclinical studies showed that the transcriptional activity of AR is modulated by signaling pathways involving FOXA1 [45] and GATA3 [12]. There have been no clinical studies in TNBC patients that have investigated the correlation of the transcript levels of these markers with disease prognostication. We have investigated the co-expression of FOXA1 and GATA3 at different expression levels of AR mRNA and found a strong correlation beyond a minimum threshold (1fold). This clearly indicates that with an increase in AR mRNA transcripts there was an upregulation of FOXA1 and GATA-3 and vice versa. In the subgroup analysis of AR positive tumors, we found co-expression of FOXA1 and GATA3, supporting the earlier reports of a possible crosstalk among all these molecules in the nuclear co-localization of AR [46, 47]. This argument is further strengthened from the finding that, patients positive for all three markers experienced a further reduction in the DMFS compared to only AR (+) patients. This important finding suggests the significant role of the co-regulators in the AR mediated tumor progression in AR (+) TNBC patients. In a recent study, AR+/FOXA1+ mRNA expression in fresh biopsy specimens has been correlated with poor prognosis of TNBC patients [9]. However, the prognostic role of GATA3 in association with AR had not been established. This is the first report demonstrating a significant association of AR along with their co-regulators in the prognosis of TNBC patients. Moreover, findings of uni and multivariate Cox analyses with different expression levels of AR strengthen its importance as a strong predictor of distant metastasis, though these results need to be validated in a larger cohort. These findings have important clinical significance in identifying a subpopulation of AR (+) tumors, which can be targeted for anti-AR therapies.

## Conclusion

The results of this study indicate that TNBC comprises a subset of AR (+) tumors with luminal marker expression. qRT-PCR is a sensitive method to detect low transcript copies of all these markers in tissues which lacks protein expression. AR mRNA expression accompanied by its co-regulators, FOXA1, and GATA3 indicates a luminal phenotype. These data also indicate AR expression itself is an independent marker for poor prognosis in TNBC. Concurrent evaluation of AR and its co-regulators suggests that the three-marker combination of AR/FOXA1/GATA3 could be superior to AR alone as a prognostic marker. However, this requires further examination in a larger cohort since the AR/FOXA1/GATA3 cases are a small subset of AR (+) cases in the current cohort. Therefore, AR positivity in TNBC patients offers alternative targeted therapy options for better management of the disease.

## Supplementary information

**Additional file 1 **: **Table S1**. Primer and probe sequence of all the genes in the study. **Table S2**: FOXA1 and GATA-3 co-expression at different threshold of AR mRNA. **Table S3**: Comparison of AR qRT-PCR positive cases with AR Protein Expression by IHC. **Table S4**: Concordance between AR protein expression and AR mRNA at different thresholds of expression. **Table S5**: Univariate and Multivariate Analysis using Cox proportional hazard method

**Additional file 2 **: **Figure S1**: Analysis of assay efficiency: PCR efficiency of each gene was assessed using 10- point serial dilutions (log2) (1:2 with nuclease free water) of cDNA generated from a pooled RNA test sample. Starting concentration for GAPDH, RPLPO and FOXA1 assays was 2^^3^ and that of target assays was 2^^6^. Efficiencies of GAPDH and RPLPo (a, b); Efficiencies of FOXA1, AR. Ki67 and GATA-3 respectively (c, d,e, f). The assay was considered linear if the deviation from linearity, i.e. the difference between the best and the linear regression model, did not exceed 1 Cq value. The efficiency was calculated for each gene using the formula mentioned in supplementary methods and was ranging from 87.14 to 106%. **Figure S2**: Concordance between Ki67 protein and mRNA Expression. IHC was performed on 21 cases which were also positive for AR by qRT-PCR. The bar graph of the correlation analysis indicated a 62% concordance between the two methods. **Figure S3**: Effect of AR protein and different AR mRNA threshold levels in TNBC prognosis. (a) The distant metastasis free analysis of patients with (+) and (−) AR protein expression by IHC. (b) DMFS of TNBC patients stratified based on AR low/no (< 1) vs high (> 10.0) mRNA levels.

**Additional file 3: Supplementary method.** Primer and probe effciency study.

## Data Availability

The datasets used and/or analyzed during the current study are available from the corresponding author on reasonable request.

## Abbreviations

TNBC: Triple- Negative Breast Cancer
LAR: Luminal Androgen Receptor
AR: Androgen Receptor
IHC: Immunohistochemistry
DMFS: Distant Metastasis Free Survival
qRT-PCR: quantitative Real Time PCR
NACT: Neo Adjuvant Chemotherapy
IDC: Invasive Ductal Carcinoma
EC: Ethical Committee
Cq: Cycle quantification Threshold
CCI: Cell Conditioning Solution
DAB: 3-3'Diaminobenzidine
