# Supplementary material for: Androgen Receptor mRNA levels determine the prognosis in triple-negative breast cancer patients
Source: BMC Cancer. 2020 Aug 10;20:745. doi: 10.1186/s12885-020-07218-0 (PMC7419184; doi:10.1186/s12885-020-07218-0)
Supplement: Supplementary file 1 — Additional file 1 : Table S1. Primer and probe sequence of all the genes in the study. Table S2: FOXA1 and GATA-3 co-expression at different threshold of AR mRNA. Table S3: Comparison of AR qRT-PCR positive cases with AR Protein Expression by IHC. Table S4: Concordance between AR protein expression and AR mRNA at different thresholds of expression. Table S5: Univariate and Multivariate Analysis using Cox proportional hazard method [file 12885_2020_7218_MOESM1_ESM.pdf]

Table S1: Primer and Probe Sequences used in the study

**Gene Name**	**Sequence of the Primer 5' to 3'**
GAPDH -Forward	GACAGTCAGCCGCATCTTCT
GAPDH- Reverse	GCGCCCAATACGACCAAATC
GAPDH- Probe	CGTTGACTCCGACCTTCACCTTCC
RPLPo Forward	CGACCTGGAAGTCCAACTAC
RPLPo Reverse	GCTTGGAGCCCACATTGTCT
RPLPo Probe	TCCGAAATGTTTCATTGTGGGAGC
Ki67 Forward	CCACACTGTGTCGTCGTTTG
Ki67 Reverse	CCGTGCGCTCATCCATTCA
Ki67 Probe	CCTATGTCCTCCAGGGCACGGTGG
FOXA1 Forward	GTGAAGATGGAAGGGCATGAA
FOXA1 Reverse	CCTGAGTTCATGTTGCTGACC
FOXA1 Probe	AACAGCTACTACGCAGACACGCAG
**Assay Name**	**IDT Predesigned Assay ID**
AR	Hs.PT.56a.40798740
GATA-3	Hs.PT.58.4308511

Table S2: FOXA1 and GATA3 co-expression at different expression cut off of AR mRNA

Cut off (AR)	No of AR (+) cases	FOXA1 low	FOXA1high	GATA3 low	GATA3 high
**0.02-1**	12	6/11(55%)	5/11 (45%)	9/11 (82%)	2/11(18%)
**>1-10**	18	2/15 (13.0%)	**13/15 (87%)**	5/15 (33%)	**10/15 (77%)**
**>10.0**	4	1/4 (25%)	**3/4 (75%)**	1/4 (25%)	**3/4 (75%)**

Table S3: Comparison of AR qRT-PCR positive cases with AR Protein Expression by IHC

Sl No	Outcome	AR + qRT	AR by IHC
1	Rec	Negative	40%
2	Rec	Positive	85%
3	Rec	Positive	70%
4	Non-Rec	Positive	90%
5	Non-Rec	Negative	10%
6	Non-Rec	Positive	95%
7	Non-Rec	Positive	80%
8	Rec	Positive	65%
9	Rec	Positive	50%
10	Non-Rec	Negative	10%
11	Non-Rec	Positive	10%
12	Non-Rec	Positive	25%

Note: Rec: Recurrence, Non-Rec: No Recurrence; Positive: AR amplified by qRT-PCR and Negative: No amplification of AR by qRT-PCR

Table S4: Concordance of AR protein and mRNA expression at different threshold of AR mRNA

Cut off (AR mRNA)	Counts	AR IHC (+)	AR IHC (-)	IHC not done
**<1.0 fold**	10	3 (30%)	7 (70%)	2
**>1-10 fold**	15	5 (33%)	10 (56%)	3
**>10.0 fold**	4	2 (100%)	0 (0%)	2

Table S5. Univariate and Multivariate Analysis using Cox proportional hazard method

Parameters	Univariate			Multivariate		
	HR	95%CI	P-value	HR	95%CI	P-value
**Age (>60 vs. 40-60 and <40)**	1.99	1.09-3.61	**0.02**	Not significant		
**Grade 3 vs. 2**	0.41	0.21 – 0.8	0.008	Not significant		
**Tumor Stage III vs. I-II**	5.3	2.7 – 9.9	**<0.0001**	7.3	1.1-48.35	**0.0387**
**AR Pos vs. Neg**	1.1	0.57 – 2.4	0.63	Not significant		
**AR High vs. Low**	6.6	1.4-31.17	**0.0164**	8.47	1.57-45.49	**0.0127**
**FOXA1 High vs. Low**	1.3	0.54-3.4	0.5	Not significant		
**GATA3 high vs. Low**	2.0	0.82-4.85	0.12	Not significant		
