# Supplementary figures and images for: Androgen Receptor mRNA levels determine the prognosis in triple-negative breast cancer patients
Source: BMC Cancer. 2020 Aug 10;20:745. doi: 10.1186/s12885-020-07218-0 (PMC7419184; doi:10.1186/s12885-020-07218-0)

Figure S1

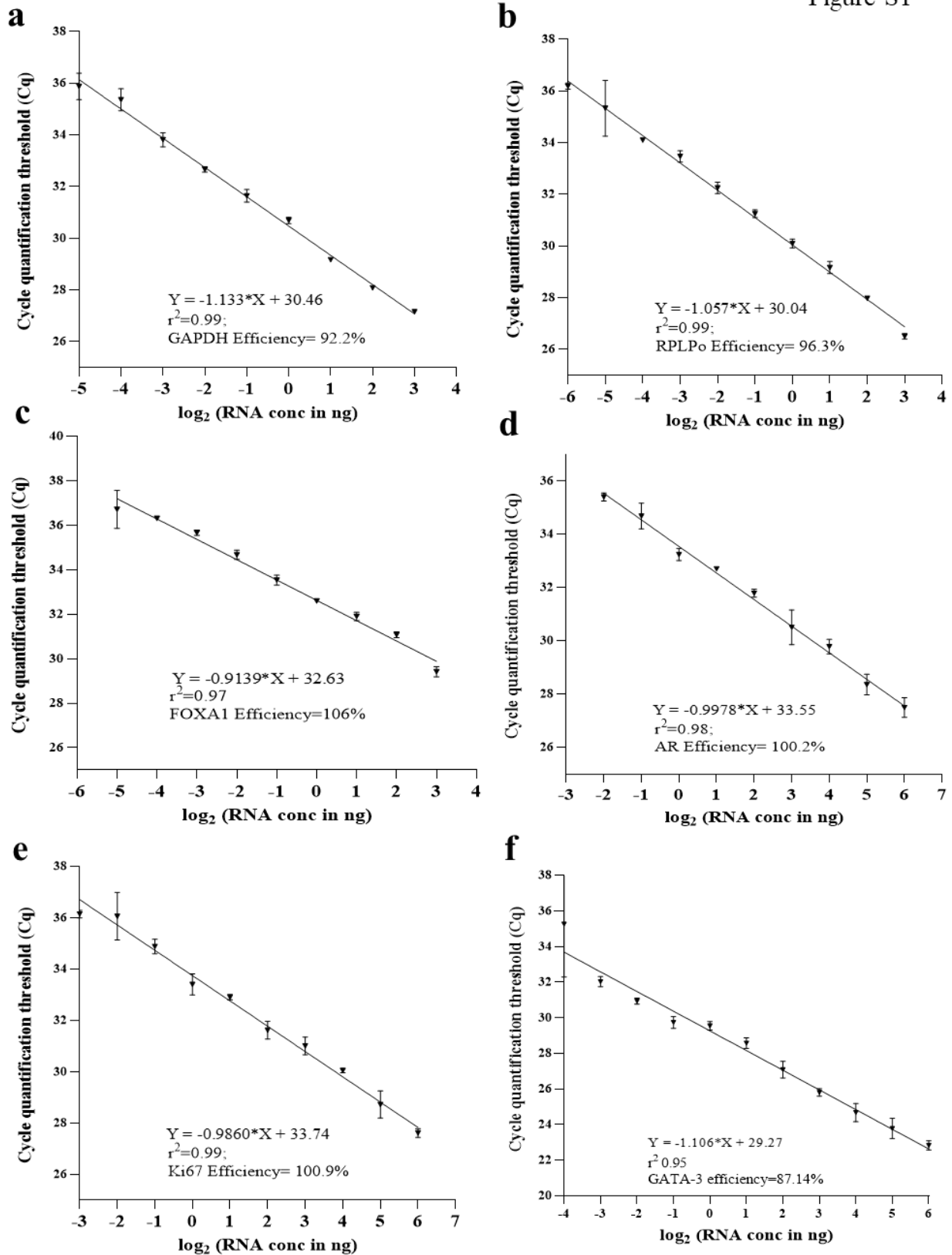

**Figure S2**

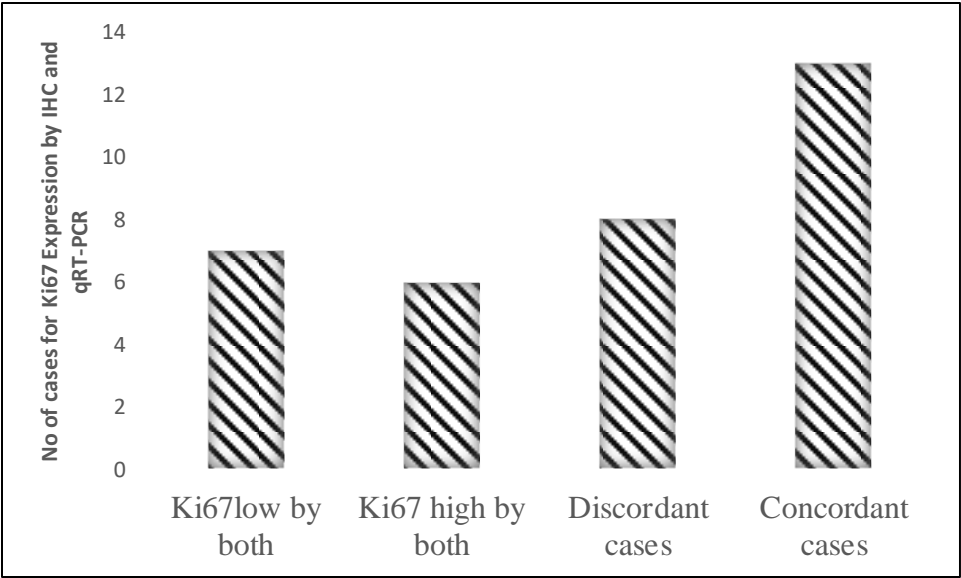

Figure S3

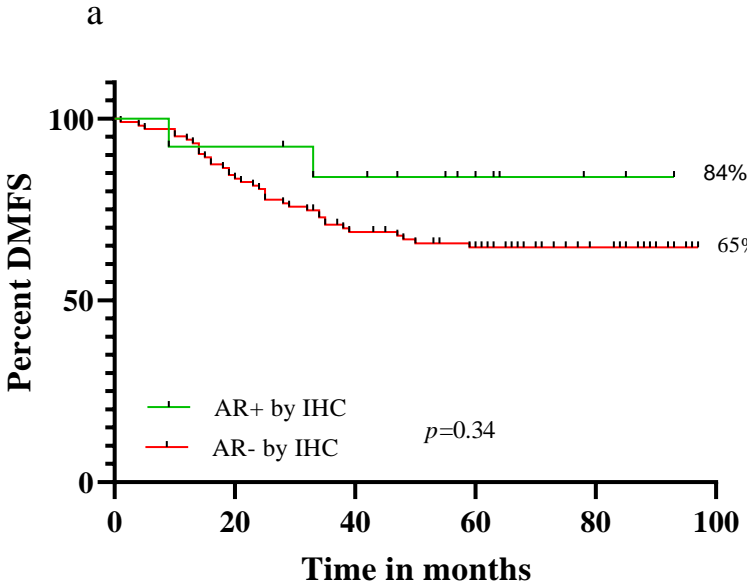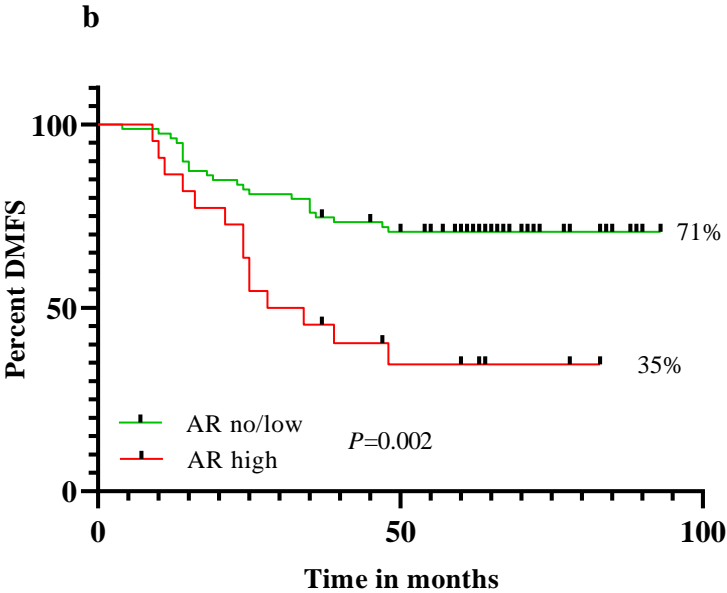

Supplement: Supplementary file 2 — Additional file 2 : Figure S1: Analysis of assay efficiency: PCR efficiency of each gene was assessed using 10- point serial dilutions (log2) (1:2 with nuclease free water) of cDNA generated from a pooled RNA test sample. Starting concentration for GAPDH, RPLPO and FOXA1 assays was 2^3 and that of target assays was 2^6. Efficiencies of GAPDH and RPLPo (a, b); Efficiencies of FOXA1, AR. Ki67 and GATA-3 respectively (c, d,e, f). The assay was considered linear if the deviation from linearity, i.e. the difference between the best and the linear regression model, did not exceed 1 Cq value. The efficiency was calculated for each gene using the formula mentioned in supplementary methods and was ranging from 87.14 to 106%. Figure S2: Concordance between Ki67 protein and mRNA Expression. IHC was performed on 21 cases which were also positive for AR by qRT-PCR. The bar graph of the correlation analysis indicated a 62% concordance between the two methods. Figure S3: Effect of AR protein and different AR mRNA threshold levels in TNBC prognosis. (a) The distant metastasis free analysis of patients with (+) and (−) AR protein expression by IHC. (b) DMFS of TNBC patients stratified based on AR low/no (< 1) vs high (> 10.0) mRNA levels. [file 12885_2020_7218_MOESM2_ESM.pdf]
