# Supplementary material for: Androgen Receptor mRNA levels determine the prognosis in triple-negative breast cancer patients
Source: BMC Cancer. 2020 Aug 10;20:745. doi: 10.1186/s12885-020-07218-0 (PMC7419184; doi:10.1186/s12885-020-07218-0)
Supplement: Supplementary file 3 — Additional file 3: Supplementary method. Primer and probe effciency study. [file 12885_2020_7218_MOESM3_ESM.docx]

Supplementary Method

**Measurement of primer efficiency and linearity**

The linear response and amplification efficiency of primers/probes for AR, GATA-3, Ki67, FOXA1, GAPDH and RPLPo was obtained from serial dilution experiments. For this, a test RNA was made from 15 pooled RNA samples to determine the limit of detection as a function of performance characteristics of each gene. Approximately 1ug of RNA was converted into cDNA by gene specific conversion method. The cDNA equivalent of 8 ng RNA was serially diluted into 10 point two-fold dilutions and qRT-PCR was performed in triplicate reactions for individual genes. By plotting the Ct values relative to input RNA concentrations, a linear regression graph was generated. From the linear regression equation, the amplification for each gene was calculated using the equation, E= (2^-1/Slope^ − 1) x100%., where the slope is estimated from the simple linear regression of CT measurements vs log2 RNA concentration. RT-PCR analysis was done twice with triplicate assays to generate 6 data points for each dilution. Mean CT scores from both runs were averaged to assign a final expression value for each RNA concentration measurement used in the linear regression analysis.
